# Cluster Headache Presenting As Sinonasal Pathology in a Young Adult: A Diagnostic Odyssey

**DOI:** 10.7759/cureus.53199

**Published:** 2024-01-29

**Authors:** Sanya Gupta, Ashish Anjankar, Chirag Sathe

**Affiliations:** 1 Neurology, Jawaharlal Nehru Medical College, Datta Meghe Institute of Higher Education and Research, Wardha, IND; 2 Biochemistry, Jawaharlal Nehru Medical College, Datta Meghe Institute of Higher Education and Research, Wardha, IND; 3 Microbiology, Jawaharlal Nehru Medical College, Datta Meghe Institute of Higher Education and Research, Wardha, IND

**Keywords:** headache disorders, neurology and systemic disease, cluster headache, woman health in neurology, chronic cluster headache

## Abstract

Cluster headache is a debilitating primary headache disorder marked by severe, unilateral pain often accompanied by autonomic symptoms. We describe the case of a 20-year-old student who presented with excruciating peri-orbital pain localized to the right side, accompanied by ipsilateral nasal obstruction, a nasal spur, and a deviated nasal septum (DNS). The initial clinical picture strongly suggested sinonasal pathology, leading to investigations and treatments aimed at this presumed diagnosis. However, as the patient's symptoms persisted and evolved over time, with episodes of recurrent and intense pain associated with ipsilateral tearing, rhinorrhea, and ptosis, further evaluation was pursued. A comprehensive assessment, including detailed headache characteristics, neurological examination, and neuroimaging, ultimately revealed the diagnosis of cluster headache. This case emphasizes the diagnostic challenges associated with atypical presentations of cluster headaches, the importance of a meticulous clinical evaluation, and the need for early recognition to provide timely and effective interventions for these severely affected individuals.

## Introduction

Cluster headache is a trigeminal autonomic cephalalgia characterized by powerful, unilateral, transitory headache episodes accompanied by autonomic symptoms ipsilaterally, agitation, or both. The severity of the illness has a significant influence on the patient's quality of life and might occasionally provoke suicidal thoughts. It is currently believed that synchronized aberrant activity in the autonomic, trigeminovascular (TGV), and hypothalamic neural systems causes cluster headaches. It seems that the hypothalamus (HT) is essential in creating a permissive condition that permits the start of an episode. On the other hand, the peripheral nervous system is probably what will be attacked [1].

First described by Wilfred Harris in 1926 as "erythroprosopalgia of Horton," cluster headache earned its name due to the tendency of these attacks to occur in clusters or bouts. These bouts are typically marked by daily or near-daily occurrences of headaches, lasting anywhere from 15 minutes to three hours, over weeks to months. They are often accompanied by autonomic manifestations such as ptosis, miosis, tearing, rhinorrhea, and facial sweating [2,3]. Cluster headache primarily affects males in their third or fourth decade of life, with a male-to-female ratio of approximately 3 to 1. Its prevalence is relatively low, estimated at 0.1% to 0.2% in the general population, making it one of the rarer headache disorders [4,5]. Despite their rarity, cluster headaches' profound impact on the quality of life of those afflicted cannot be overstated. The intensity and frequency of the attacks often lead to significant disability, impair daily activities, and cause social and occupational disruption [6].

While the pathophysiology of cluster headaches remains incompletely comprehended, recent advances in neuroimaging and neurobiology have shed light on potential mechanisms, including hypothalamic involvement and trigeminal nerve activation [7]. The management of cluster headaches poses a significant clinical challenge due to the rapid onset and intensity of the pain, necessitating prompt and effective interventions.

In this case report, we propose a diagnostically challenging case of a 20-year-old student initially misdiagnosed with sinonasal pathology and ultimately diagnosed with cluster headaches. This case underscores the importance of recognizing atypical presentations of cluster headaches to ensure timely intervention and alleviate the substantial burden it places on patients' lives.

## Case presentation

A 20-year-old female presented to the OPD with chief complaints of episodes of extremely decapacitating right-sided periorbital pain associated with rhinorrhea and photophobia, nasal congestion on the same side, and increased lacrimation on the same side. The patient was first referred to an ophthalmologist due to her history of congenital myopia with refractive power of -6 D in the left eye and -6.5 D in the right eye. After effectively changing spectacles and receiving no consecutive relief, the patient came to the ENT OPD with the same complaints. CT and general systemic examination revealed a nasal spur in the anterior septum, pressing on the lateral side of the nose, which caused acute sinusitis, which was ruled to cause pressure headaches.

The patient was then treated for seemingly recurring sinusitis, and the analgesics given temporarily relieved the patient of the pain. However, after the due course of medication was completed, the patient again complained of periorbital pain, this time with increased duration and frequency, and thus was referred to neurology. With the classic manifestations of three to four episodes of excruciating, mostly right-sided and sometimes left-sided periorbital pain radiating to the temple and jaw, nasal congestion of the same side, rhinorrhoea, and increased lacrimation on the same side, with each episode lasting 15-30 minutes, the patient was diagnosed with cluster headache. Triggers noted for the same were increased caffeine consumption, direct work in sunlight, and stress.

Investigations

**Figure 1:**
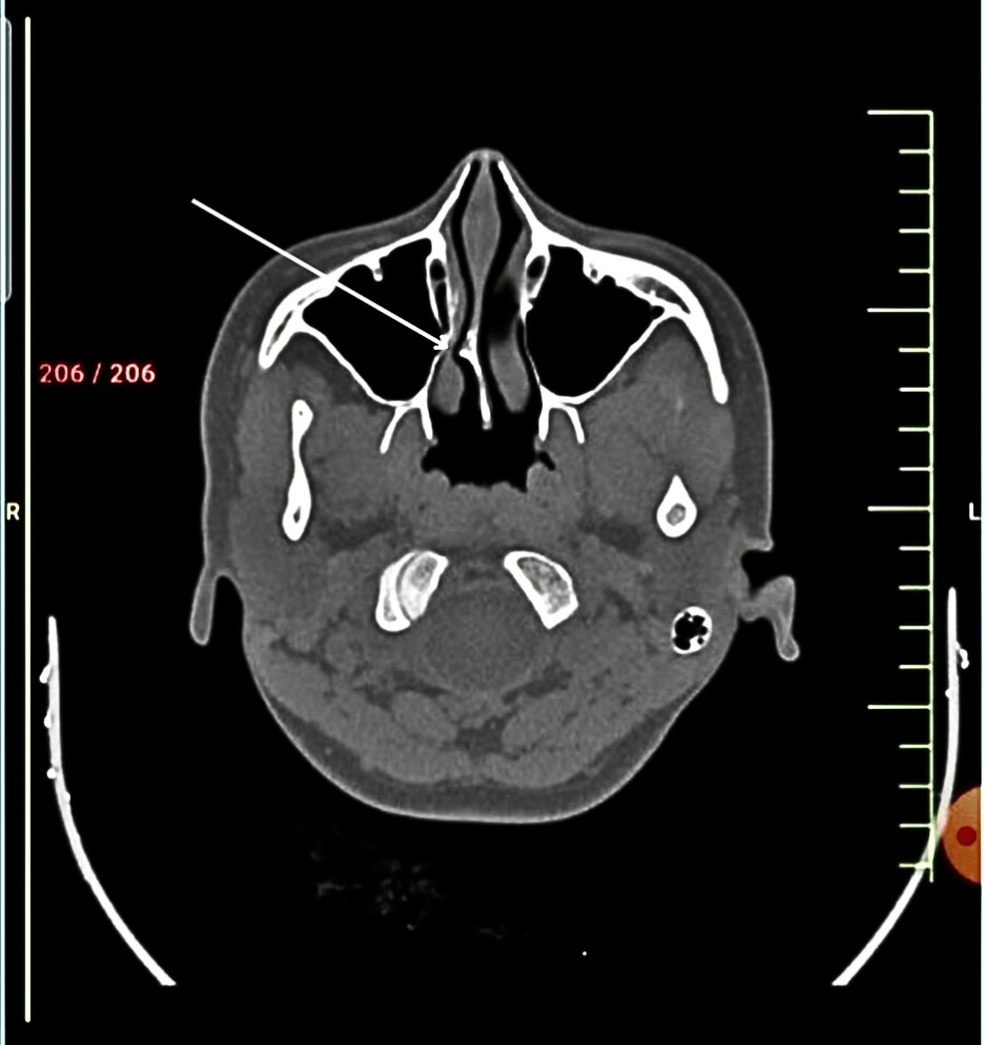
CT scan of the paranasal sinuses of the patient showing anterior nasal septum spur on the right side

The patient went to different departments, each with investigations of their own. Ophthalmoscopy was done to rule out pain due to changes in refractive power. A CT of the paranasal sinus was done, with images of the result attached below. The patient was found to have a right-sided deviated nasal septum (DNS) in the form of an anterior spur. Figure 1 corresponds to the patient’s CT scan of paranasal sinuses showing an anterior nasal spur in the right nasal cavity, presenting as recurrent sinusitis, with symptoms of rhinorrhoea, nasal stuffiness, and pressure headaches.

**Figure 2:**
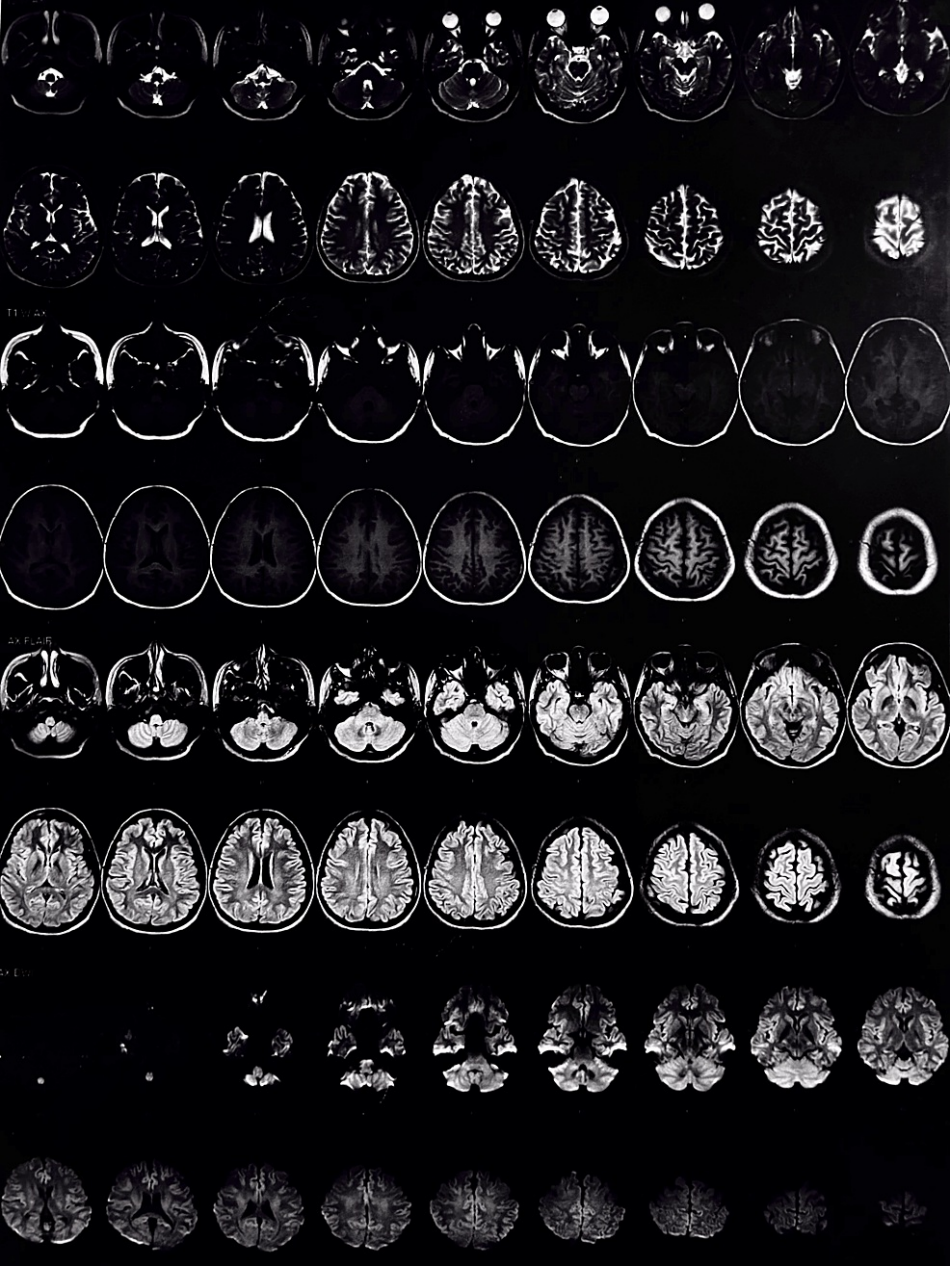
MRI scan of the patient showing no deformities and normal cerebral activity

An MRI of the brain was done to rule out any other causes, the results of which are attached below. The scan results came to be expected; no defects or deformities were seen. Figure 2 shows the patient’s MRI report with no significant abnormalities or dysfunctions.

Treatment

The patient was first put on a treatment regime for sinusitis, which included a five-day course of cefixime, antihistamines, and analgesics, due to which she experienced temporary relief, where pain later exaggerated on completion of the course. The patient then self-administered antihistaminics for one month, which, after initial relief, failed to suppress the attacks. Following the diagnosis of cluster headaches, an MRI revealed no significant changes. Thus, the patient was put on the prophylactic dosage, starting with verapamil 40 mg twice a day, amitriptyline 10 mg once every night, and gabapentine 100 mg twice a day for a month. The patient was advised to take rizatriptan benzoate 10 mg or ketorolac tromethamine 10 mg for emergency attacks. Patients with episodic cluster headaches who experience bouts lasting longer than four to eight weeks may consider preventative care. This is especially valid for those who suffer from persistent cluster headaches. Verapamil (200 to 900 mg) is the most efficacious medication with the greatest available scientific data, while lithium comes in second [6]. Additionally extremely helpful in avoiding cluster headaches are amitone and gabapentin. Verapamil was also given at a very low dosage because the patient is quite young.

After a month of this regimen, the number of attacks reduced; therefore, gabapentin was removed, and the patient was put on the same regimen for two months. Due to side effects, including constipation, the patient was switched on to duloxetine 10 mg and propranolol hydrochloride 40 mg. The patient is currently experiencing minimum, almost nil attacks and goes on to live a relatively everyday life.

Pathophysiology

**Figure 3:**
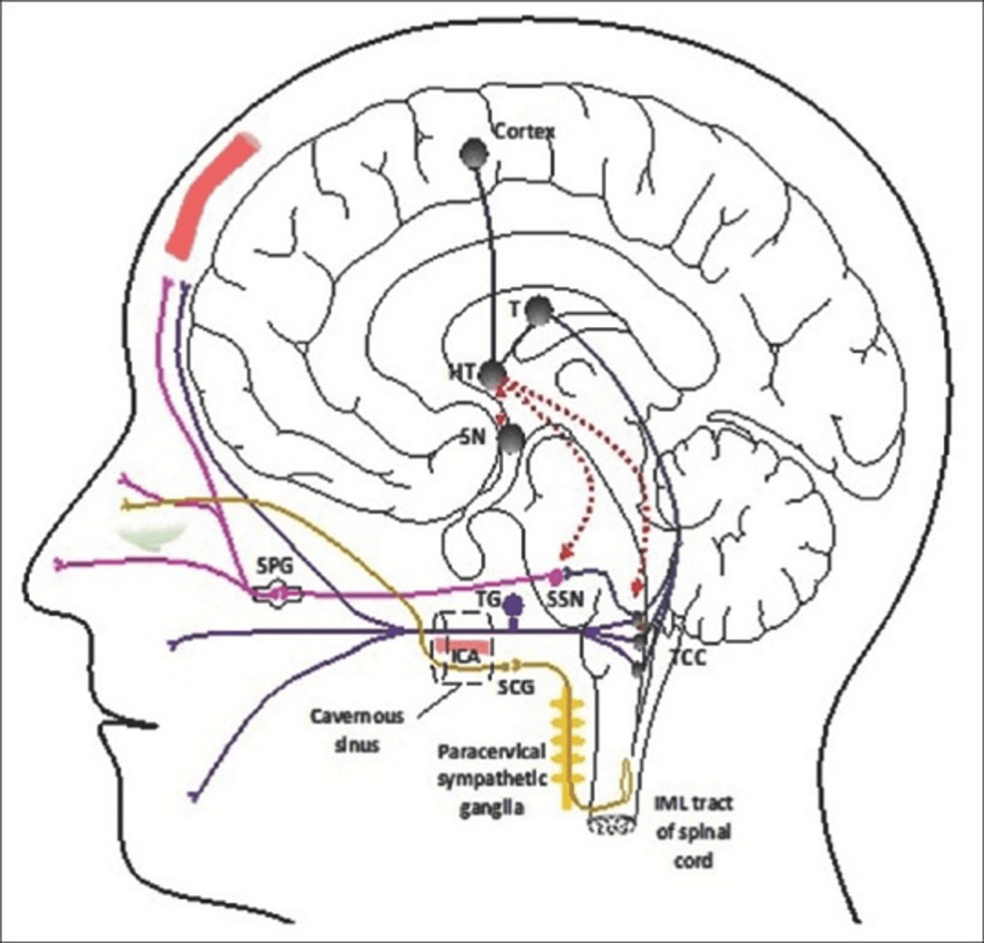
The pathophysiology of cluster headaches HT: hypothalamus, ICA: internal carotid artery, IML: intermediolateral tract of the spinal cord, SCG: superior cervical ganglion, SN: suprachiasmatic nucleus, SPG: sphenopalatine ganglion, SSN: superior salivatory nucleus, TCC: trigeminocervical complex [7]

Pain signals from the TGV system enter through the ophthalmic division of the V cranial nerve, also known as the trigeminal nerve, and get input from the cranial arteries and dura mater (shown by purple fibers). Pain perception is the result of these inputs synapsing in the trigeminocervical complex (TCC) and projecting to higher brain areas such as the cortex and thalamus, as represented by blue fibers. Stimulation of dural structures activates the TGV system, which activates the superior salivatory nucleus (SSN) in the pons, the cell source for the parasympathetic autonomic cranial vasodilator pathway. The SSN's output begins with the parasympathetic response, which is relayed via the face (VII cranial) nerve (not depicted) and the sphenopalatine ganglion (SPG) (pink fibers). The stimulation of the trigeminal and autonomic nerves establishes the autonomic trigeminal reflex arc, which plays an important role in the development of cluster headaches and other TACs. The HT is functionally linked to the ipsilateral trigeminal system and other pain-related brain regions. Red-dashed lines indicate the mechanisms by which HT causes and/or modifies pain. Yellow fibers indicate a sympathetic, third-order nerve injury that may cause sympathetic symptoms (incomplete Horner syndrome). This lesion is thought to be caused by vascular alterations to the ICA in the cavernous sinus, which irritates the adjacent plexus of nerve fibers [8]. Figure 3 illustrates the pathophysiology of cluster headaches.

The TGV pathway comprises neurons innervating the dura mater and cerebral arteries through cell bodies within the trigeminal ganglion. The ganglion, consisting of bipolar cells, has filaments synapsing within the TCC, including the trigeminal core caudalis in the caudal brainstem and the tall cervical line in the dorsal horns of C1 and C2. Synaptic connections exist between the dura mater and cerebral vessels. Cortical areas, such as the frontal cortex, insulae, and cingulate cortex, with projections from the TCC to the thalamus, are involved in processing pain. Trigeminal ganglion cell bodies contain vasodilator peptides, including substance P, neurokinin A, and calcitonin gene-related peptide (CGRP). Both unrestrained and nitroglycerin-induced cluster cerebral pain attacks elevate CGRP, indicating activation of the TGV circuit during attacks [9].

Cranial autonomic indicators typical of cluster migraine result from parasympathetic outpouring from the cranial facial nerve's predominant salivatory core via the SPG, causing vasodilation and parasympathetic activation on the trigeminal-autonomic reflex pathway. Clinical manifestations include nasal blockage, conjunctival infusion, and lacrimation. Carotid vasodilation and parasympathetic enactment occur when pain activates the trigeminal nerve's main division, as observed in capsaicin infusion. The clinical features of cluster cerebral discomfort point to the HT as a key component. Observations by Kudrow indicate that clustered cerebral pain episodes follow a circannual pattern, especially during seasonal changes. This circannual rhythm, linked to photoperiodism and the duration of daylight, is attributed mostly to the HT. The inner circannual pacemaker seems to be desynchronized with external light cues. The pineal gland, under suprachiasmatic core control, produces melatonin, maintaining a steady circadian rhythm. The HT and the mindful cervical plexus, as well as the carotid plexus, provide innervation to the suprachiasmatic core and the autonomic centers of the thoracic spinal column; elevated light serves as the most natural stimulant for daily melatonin production. The suprachiasmatic center of the HT receives information from the retina via a coordinated route. Melatonin discharge is observed to be reduced during bouts in ECH patients, with atypical excretion of melatonin metabolites blunting the characteristic evening peak. Studies, including a placebo-controlled study, case reports, and research on melatonin as an adjuvant therapy for cluster headache prevention, provide evidence of the usefulness of melatonin replacement in cluster migraine treatment. Examination of the role of other neuroendocrine hormones such as orexin, testosterone, and cortisol further demonstrates the HT's involvement in cluster cerebral pain [8-11].

Practical neuroimaging theories demonstrate the activation of the rear HT in both unrestricted cluster cerebral pain attacks and those triggered by intravenous nitroglycerin. Focusing on the rear hypothalamic gray through deep brain stimulation in patients with cluster cerebral pain further substantiates the role of the HT in cluster migraine.

## Discussion

Cluster headache, a crippling neurological condition, is characterized by persistent, excruciating, and unilateral head pain that is frequently localized around the eye. Cluster headache typically develops in middle-aged males and has a recognized clinical pattern, including recognizable autonomic symptoms [8]. This case study, however, emphasizes the difficulties in diagnosing patients who appear with atypical symptoms, which might result in an incorrect initial diagnosis and a delay in treatment. This 20-year-old student initially presented with right-sided peri-orbital pain, a right-sided nasal spur, and DNS, which closely resembled sinonasal pathology. Due to the difficulty in making a diagnosis due to this unique presentation, numerous examinations and therapies focused on the alleged sinonasal problem were conducted. According to multiple published cases in the literature, such diagnostic lag is prevalent in cluster headache cases with distinctive presentations [9].

To reduce pain and limit the adverse effects on a patient's quality of life, timely identification and treatment of cluster headaches are crucial [10]. This example emphasizes how critical clinical expertise is in identifying uncommon cluster headache presentations, especially in younger patients, to prevent pointless investigations and therapies. Additionally, it highlights the importance of a thorough examination, which includes a rigorous headache history, a neurological exam, and neuroimaging, in making the proper diagnosis. The proper pharmacological therapy was started in this situation, and the patient's needs-specific lifestyle changes were implemented for effective management. It was quickly determined that the patient had a cluster headache, which allowed for effective treatment and relief from crippling symptoms. Therefore, this research underlines that an accurate diagnosis is important to a patient's overall health.

## Conclusions

Presenting with symptoms initially resembling sinonasal pathology, a 20-year-old student underwent a diagnostic journey that ultimately revealed a diagnosis of cluster headache. This case report emphasizes the challenges of distinguishing between cluster headaches and sinonasal issues. Moreover, it underscores the importance of a comprehensive clinical evaluation that includes a detailed headache history, a neurological examination, and appropriate neuroimaging.

The significance of considering cluster headaches in differential diagnosis cannot be overstated, especially in cases where young patients present with atypical symptoms. Maintaining a high index of suspicion for cluster headaches in unusual clinical situations is imperative for healthcare professionals, as emphasized by the report. It reminds them to act promptly and with precision to improve patient quality of life. A timely and accurate diagnosis is of paramount importance in this regard.
